# Network Analysis of Symptoms of Generalized Anxiety and Autism: Discrete but Connected

**DOI:** 10.1002/jdn.70006

**Published:** 2025-02-12

**Authors:** Vicki Bitsika, Christopher F. Sharpley, Kirstan A. Vessey, Ian D. Evans

**Affiliations:** ^1^ Brain‐Behaviour Research Group University of New England Armidale Australia; ^2^ School of Science & Technology University of New England Armidale New South Wales Australia

**Keywords:** anxiety, autism, network

## Abstract

Autism spectrum disorder (ASD) is comorbid with several major psychiatric disorders, primarily anxiety. Although a previous report of a network analysis of five anxiety subtypes and some ASD diagnostic criteria suggested that anxiety was not part of the ASD symptomatology, several methodological limitations challenge the conclusions reported there. To address those limitations and extend understanding of the association between ASD and anxiety, data on ASD symptomatology and the symptoms of generalized anxiety disorder (GAD) were collected from 150 autistic boys and their parents and were analysed via network analysis. Results indicated that, although the separation of GAD and ASD symptoms was generally confirmed, several connections were found between the two sets of symptoms, arguing for a more nuanced model of the association between these two disorders. These findings hold implications for the delivery of ‘precision‐medicine’ treatment models for the treatment of anxiety in ASD.

## Graphical abstract

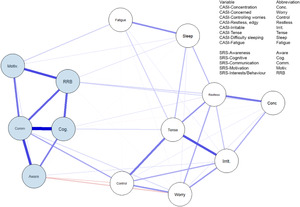

## 1 Introduction

Autism spectrum disorder (ASD) is a neurodevelopmental disorder characterized by difficulty in social interaction and communication (SC), plus restrictive and repetitive behaviours (RRBs) (APA 2022). Comorbid psychiatric disorders are also often found in autistic people (Hossain et al. 2020), predominantly anxiety disorders (Vasa et al. 2020; Jenkinson, Milne, and Thompson 2020), with prevalence ranging up to 62% (Steensel and Heeman 2017). Although some early explanations of the high prevalence of anxiety (Kerns and Kendall 2012) suggested that anxiety was an integral aspect of ASD itself, Montazeri et al. (2019) demonstrated a clear separation between the subtypes of anxiety and the core ADOS indicators of ASD, but the generalizability of that finding was restricted by a number of methodological limitations.

First, the age range of the autistic youth was narrow, consisting of just 3.5 years. Second, the issue of who is to provide the ratings of autistic youth's anxiety is also important because parents' evaluations of their child's anxiety may be distorted by their own anxiety status (Bitsika et al. 2015). Autistic boys and adolescents can rate their anxiety severity more accurately than their parents when compared with a biological indicator of stress and anxiety (Bitsika et al. 2014). Third, Montazeri et al. (2019) included a small subsample of autistic girls that comprised just over 15% of the total sample but then did not analyse the male and female data separately. A focus on males only may be appropriate at the initial stage of network analysis, particularly as further data have emerged regarding the differences in the ways that ASD manifests in females versus males (Lai et al. 2011; Rubenstein, Wiggins, and Lee 2015). Due to the reported higher prevalence rates of ASD in males than females of just over 3:1 (Loomes, Hull, and Mandy 2017), a sample of young autistic males might be the preferred initial step in further understanding how anxiety and ASD are related, but certainly when making any comparison with Montazeri et al.'s (2019) initial results. Fourth, the ADOS is a recommended instrument for clinicians to observe autistic persons' behaviour and rate its functionality, but clinicians are rarely as familiar with the autistic child's behaviour as are the parents of that child, and it may be that information. It may be that, following diagnosis as ASD via the ADOS, more detailed information about a child's ASD‐related traits could be collected from their parents. Finally, Montazeri et al. (2019) did not use measures of anxiety that included all the accepted DSM‐5‐TR diagnostic criteria for a particular anxiety disorder.

Other work has been done regarding the networks of ASD and anxiety. For example, on adults with ASD and also intellectual dysfunction (Sáez‐Suanes 2024), adult males and females with vs. without ASD (Radhoe et al. 2024), with children and teenagers with ASD and ADHD (Goldstein et al. 2001) and several studies of the ASD diagnostic criteria alone (Lee, Gau, and Tseng 2024; Wang et al. 2020). However, to date, the particular limitations in Montazeri et al.'s (2019) study have not been directly examined.

The issue of same vs. different sex sampling needs some discussion. There is sufficient data now accepted to agree that males and females differ in some important aspects of ASD (Rubenstein, Wiggins, and Lee 2015; Hull, Mandy, and Petrides 2017) and anxiety (Bangasser and Cuarenta 2021), arguably because some autistic females are able to ‘camouflage’ their autistic behaviour, particularly in regard to social interactions (Hull, Petrides, and Mandy 2020). When undertaking a major comparison across genders, males and females need to be matched on age, IQ, ASD severity and other demographic factors that may influence the expression of ASD behaviours. However, that was not the aim of this study, which instead focussed upon investigating some of the limitations from one of the first studies to report on the network analysis of ASD and anxiety—Montazeri et al. (2019)—to determine if those limitations may have confounded the overall results reported in that study.

The possible benefits of network analysis have been described in detail elsewhere (Hevey 2018; Borsboom et al. 2021) but may be briefly used as a powerful tool for uncovering the relationships and dependencies among variables. In this study, it has been applied to social responsiveness criteria in autism (SRS‐2 subscales) and anxiety subscales (CASI‐4 GAD) to provide insights into the structure and dynamics of these complex characteristics. Standard network analysis using Spearman correlation identifies associations between variables, while extended Bayesian information criterion with a graphical lasso algorithm (EBICglasso) refines these associations by selecting only the most robust connections, minimizing noise and overfitting by evaluating the data set as a whole. Together, these methods were used to help reveal the underlying network structure between SRS‐2 subscales in autism and anxiety.

Therefore, to address some of these limitations upon the findings reported by Montazeri et al. (2019) and to extend that line of research by focusing upon individual GAD symptoms instead of total scores, this study recruited a sample of autistic males aged 6–18 years (referred to as ‘boys’ from here on), all of whom had received a diagnosis of ASD. Parents provided information about their sons' autistic symptoms, but the autistic boys rated their own GAD symptoms. GAD is heterogeneous, with the eight diagnostic criteria each describing a different aspect of overall anxiety, and therefore a network analysis that includes each of those eight GAD symptoms can provide a more detailed account of the association between the core symptoms of ASD and the various symptoms of GAD. The major aim of this study was to describe the networks between ASD and GAD symptoms within a sample of young autistic males, with a view to inform a better understanding of how these two disorders are related and how to base potential clinical treatments on that relationship.

## 2 Methods

### 2.1 Participants

A priori power analysis indicated that, to detect a medium strength correlation of 0.3 or above with *p* < 0.05 and a power of 0.95, a sample of 111 was required. The current sample was drawn from a major study of the way that ASD and anxiety are related in autistic youth (Bitsika and Sharpley 2016) so that it included 150 autistic boys (*M* age = 11.2 years, SD = 3.3 years, range = 6–18 years), plus one of their parents. Although not planned, all of these parents were mothers. These participants were recruited from a local parent support group and ASD service organizations on the Gold Coast, Queensland, Australia, for a study about ‘how you think about things’. All participants were Anglo‐Saxon in ethnicity, and over 97% had been born in Australia.

All the autistic boys had received an ADOS‐2 score of at least seven to ensure that they met the criteria for ASD, plus a Full Scale IQ of above 70 on WASI‐II (Wechsler 2011), allowing them to be classified as minimally impaired in their local setting. None of the autistic boys had any concurrent genetic or neurological conditions.

### 2.2 Instruments

The Autism Diagnostic Observation Schedule Second Edition (ADOS‐2) (Lord et al. 2012). The ADOS‐2 is recommended for the purpose of classifying ASD severity (Filipek et al. 1999; National Research Council 2001) and has demonstrated sensitivity of 0.89–0.92, specificity between 0.81 and 0.85 (Lebersfeld et al. 2021) and test–retest reliability of 0.71–0.89 (Janvier et al. 2022) in previous studies.

The Child and Adolescent Symptom Inventory 4th revision (CASI‐4). The CASI‐4 uses the DSM‐5‐TR diagnostic criteria for children and adolescents (Gadow et al. 2002). The CASI‐4 has been used in studies of autistic children (Gadow et al. 2005). Psychometric data are satisfactory and include test–retest reliability of *r* = 0.67 (*p* < 0.001) over a 6‐week period and internal consistency of 0.74 (Gadow and Sprafkin 2010). Participants may respond to the CASI‐4 questionnaire items with a self‐assessment of 0 (*never*), 1 (*sometimes*), 2 (*often*) or 3 (*very often*), thus providing a measure of severity beyond that from categorical assessment procedures. The CASI‐4 has several anxiety subscales, one of which measures the eight diagnostic indicators of generalized anxiety disorder (GAD) in children as described in the DSM‐5‐TR (APA 2022), and which is called CASI‐4‐GAD here. The CASI‐4‐GAD includes such items as ‘I have difficulty controlling my worries’ and ‘I feel extremely tense and unable to relax’. The CASI‐4 authors reported a Cronbach's alpha for internal consistency of 0.74, a strong specificity of 0.90 (Gadow and Sprafkin 2010) and significant (*p* < 0.001) discriminant validity between a clinical and normative sample for the GAD subscale (Gadow and Sprafkin 2002). A major review of 36 scales for assessing anxiety in autistic youth listed the CASI‐4‐GAD among those with ‘good coverage for ASD’ (Grondhuis and Aman 2012, 1345).

The Wechsler Abbreviated Scale of Intelligence (2nd edition) (WASI‐II) (Wechsler 2011). The WASI‐II is a screening test of intelligence that possesses strong validity with the WISC‐IV. It contains four subtests with average reliability coefficients of between 0.92 and 0.96. The WASI‐II provides a quick and accurate estimate of full‐scale intelligence that is useful for research screening purposes.

The Social Responsiveness Scale (2nd ed.) (SRS‐2). The SRS‐2 (Constantino and Gruber 2012) consists of 65 items that measure the domains of interpersonal behaviour, communication and repetitive/stereotypic behaviour based on the diagnostic criteria for ASD (APA 2022) in children between 4 and 18 years of age. These domains are described in five subscales (Social Awareness, Social Cognition, Social Communication, Social Motivation, Restricted Interests and Repetitive Behaviour [RRB]). Caregivers answer the SRS‐2 items about a familiar child, using responses ranging from 0 (*not true*), 1 (*sometimes true*), 2 (*often true*) and 3 (*almost always true*) so that high scores on these five subscales indicate greater difficulties in the relevant domains. Construct validity for the SRS‐2 was reported for studies of almost 8000 cases conducted by the scale authors and confirmed by subsequent studies. For example, Bruni (2014) confirmed content validity across six relevant professional groups, and receiver operating characteristics indicated specificity and sensitivity of 0.92. The SRS‐2 has a Cronbach's alpha in excess of between 0.76 and 0.91; similar data are available for the five subscales, which range from 0.76 to 0.91 (median = 0.85).

### 2.3 Procedure

ADOS‐2, WASI‐II and CASI‐4‐GAD data were collected in the participants' homes by a research‐trained assistant to reduce the likelihood of excessive anxiety that could occur in an unfamiliar environment. Written and verbal instructions, plus modelling of the scales, were provided to each parent–son pair in their homes prior to the day of data collection and checked when the data were collected the day after they had been completed by the boys and their parents. All these procedures were approved by the Bond University Human Research Ethics Committee in accordance with the Helsinki Declaration of 1964 (Approval No. RO1516, 15 September 2013). Written informed consent to participate was provided by the autistic boys' mothers, and the boys also gave written or verbal assent to participate, depending on their age.

### 2.4 Statistical Analysis

Data were analysed via IBM SPSS 28 for mean, SD, 5% trimmed mean, ranges, skewness and kurtosis values, and normality was assessed by the Kolmogorov–Smirnov statistic. Testing for the possible confounding effects of age, IQ and ADOS‐2 score upon the CASI‐GAD and SRS‐2 subscale scores was undertaken by using the criterion of a medium association of a correlation coefficient of at least 0.3 (Cohen 1988).

Network analysis was undertaken using RStudio to form an initial Spearman correlation matrix for the network structure. This was regularized using the EBICglasso from the *bootnet* package for network estimation. Nodes were defined by the five SRS‐2 scores and the eight CASI‐4 GAD items for the 150 autistic boys. In the network, edges are the connections between nodes based on partial correlations between any two nodes, and after controlling for all other nodes in the network, their strength is depicted by edge weights. Bootstrapping using 2500 samples was used to generate 95% confidence intervals and significant differences for the edge weights, which were plotted to show the unique influence of one node on another while controlling for all other nodes.

## 3 Results

### 3.1 Data

Table 1 presents the descriptive data for the sample of autistic boys. The 5% trimmed means were very close to the actual means, suggesting that outliers were not a major source of confound. Four of the five SRS‐2 subscale scores (except Social Awareness) showed evidence of non‐normality by the Kolmogorov–Smirnov statistic, and so Spearman correlation coefficients were used to describe the associations between age, WASI‐II FS IQ, ADOS‐2 total scores, the five SRS‐2 subscale T‐scores and the CASI‐4 GAD total score. Apart from expected meaningful correlations between the ADOS‐2 total score and the five SRS‐2 subscale scores, there were no instances of *ρ* ≥ 0.3 between age, IQ, and ASD severity and the CASI‐GAD symptoms, nor between age or IQ and the five SRS‐2 subscales.

**TABLE 1:** Scale scores for 150 autistic boys*.*

Scale/score	Mean	SD	5% trimmed mean	Range
ADOS‐2 Total SA + RRB score	9.0	2.7	8.7	7–23
WASI‐2 Full Scale	94.9	12.41	94.5	73–132
SRS‐2: Social awareness T score	67.6	12.1	67.8	30–90
SRS‐2: Social cognition T score	74.9	11.9	75.6	36–95
SRS‐2: Social communication T score	74.5	12.1	73.4	22–90
SRS‐2: Social motivation T score	70.3	13.6	70.6	38–90
SRS‐2: RRB T score	76.9	12.2	77.7	45–90
CASI‐4 GAD total	9.8	4.5	9.7	0–23

Abbreviations: ADOS‐2 = Autism Diagnostic Observation Scale (2nd ed.); RRB = restricted and repetitive behaviour; SRS‐2 = Social Responsiveness Scale (2nd ed.); WASI‐2 = Wechsler Abbreviated Scale of Intelligence (2nd ed.).

### 3.2 Network Analysis

**FIGURE 1:**
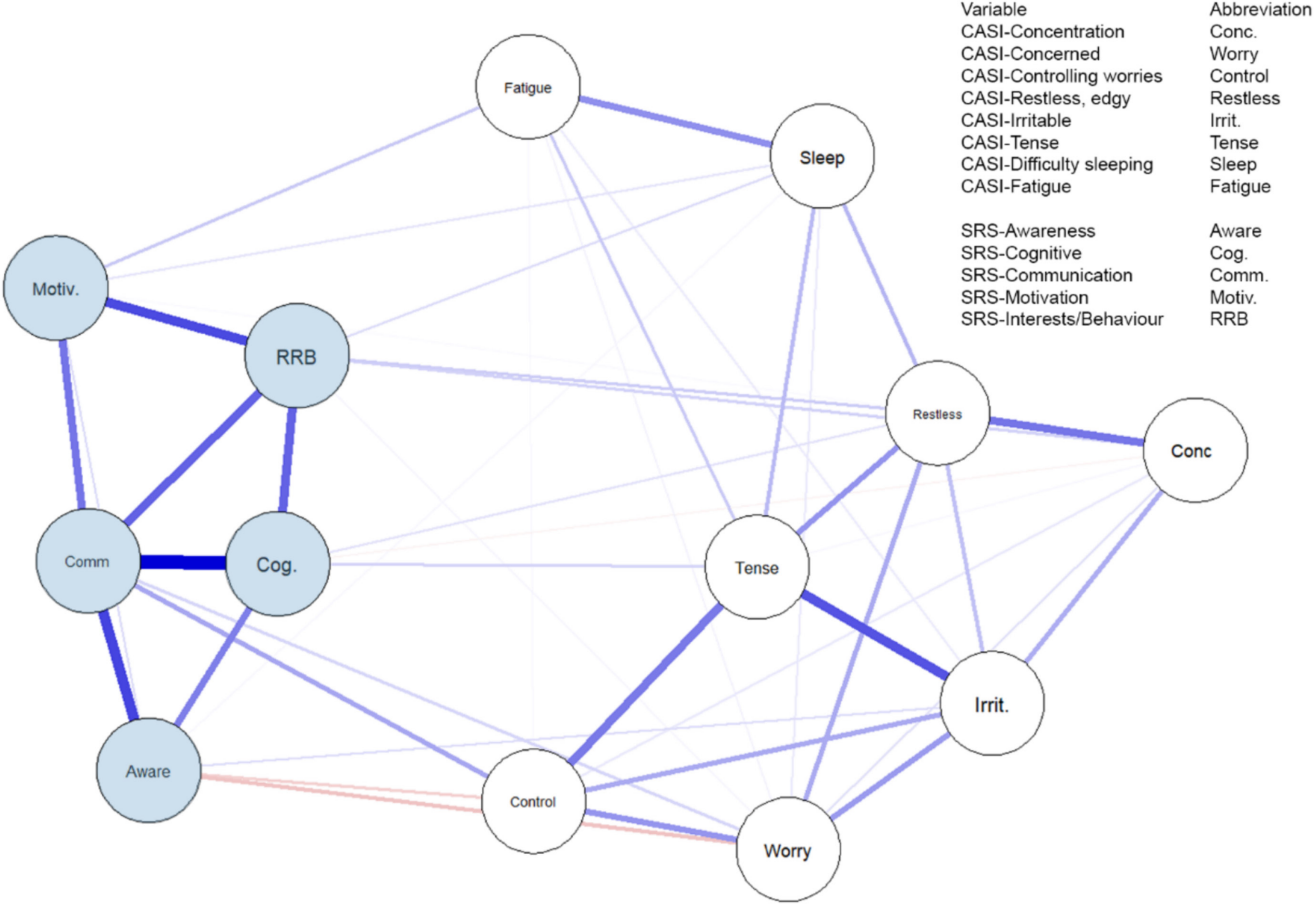
Network structure between Social Responsiveness Scale (2nd ed.) (SRS‐2) subscales and Child and Adolescent Symptom Inventory (4th ed.) Generalised Anxiety Disorder (CASI) items for 150 autistic boys. Network connections between nodes are drawn as lines where blue lines represent positive correlations and red lines represent negative correlations. The darker and thicker the line the stronger the connection. The highest strength connections are shown at the left of the plot. The metrics compared in the network analysis are CASI‐4 GAD Difficulty Concentrating (Conc.), CASI‐4 GAD Feeling Concerned (Worry), CASI‐4 GAD Difficulty Controlling Worries (Control), CASI‐4 GAD Feeling Restless or Edgy (Restless), CASI‐4 GAD Feeling Irritable (Irrit.), CASI‐4 GAD Feeling Tense (Tense), CASI‐4 GAD Difficulty Sleeping (Sleep), CASI‐4 GAD Feeling Fatigued (Fatigue), SRS‐2 Social Awareness (Aware), SRS‐2 Social Cognition (Cog.), SRS‐2 Social Communication (Comm), SRS‐2 Social Motivation (Motiv.), and SRS‐2 Restricted Interests and Repetitive Behaviour (RRB).

As may be seen in Figure 1, the SRS‐2 subscales displayed the strongest network connections with each other, and the CASI‐4 GAD items were also highly positively self‐connected. Associations between the SRS‐2 subscales and the CASI‐4 GAD items are also depicted in Figure 1.

**FIGURE 2:**
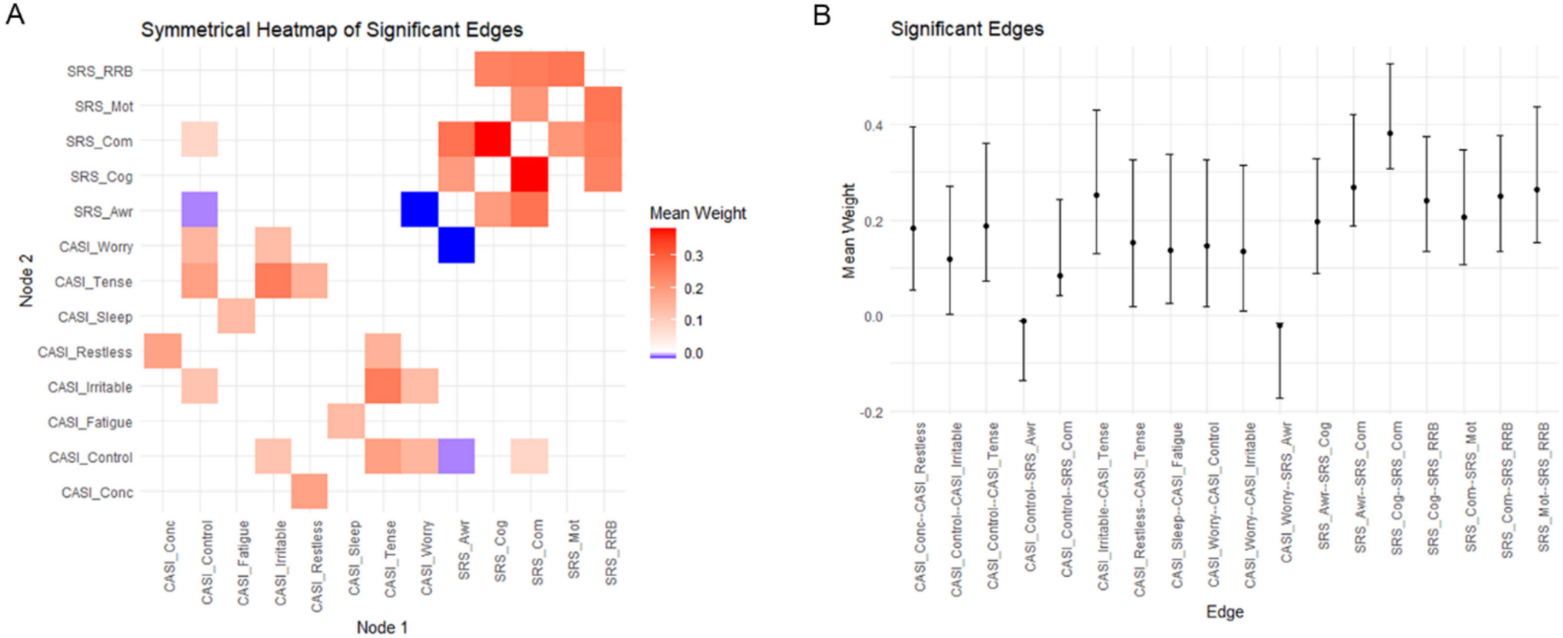
Significant network connections from the five SRS‐2 subscales and eight CASI‐4 GAD items. (A) Heatmap showing only significant edges, which represent statistically significant connections between metrics from the SRS‐2 subscales and CASI‐4 GAD items. Positive network connections are shown in red and negative in blue. (B) Edge weight means for significant network connections presented as dots with error bars showing 95% confidence intervals. The metrics compared in the network analysis are CASI‐4 GAD Difficulty Concentrating (Conc.), CASI‐4 GAD Feeling Concerned (Worry), CASI‐4 GAD Difficulty Controlling Worries (Control), CASI‐4 GAD Feeling Restless or Edgy (Restless), CASI‐4 GAD Irritable (Irrit.), CASI‐4 GAD Feeling Tense (Tense), CASI‐4 GAD Difficulty Sleeping (Sleep), CASI‐4 GAD Feeling Fatigued (Fatigue), SRS‐2 Social Awareness (Aware), SRS‐2 Social Cognition (Cog.), SRS‐2 Social Communication (Comm.), SRS‐2 Social Motivation (Motiv.), and SRS‐2 Restricted Interests and Repetitive Behaviours (RRB).

A heatmap of significant edges (significant connections between nodes after controlling for all other nodes in the network) and values for significant edge correlations (± 95% confidence interval) are presented in Figure 2A,B, respectively. As was seen in the network plot (Figure 1), SRS‐2 subscale scores correlated most strongly with each other, and the SRS‐2 score for Social Communication displayed the greatest number of significant connections. Many of the CASI‐4 GAD items were positively correlated with other CASI‐4 GAD items, with *Feeling Tense* connecting strongly with *Irritability* but also with *Feeling Restless* and an *Inability to Control Worries*. The edges for CASI‐4 GAD scores for *Trouble Sleeping* and *Fatigue* also correlated positively with each other but only weakly with any other CASI‐4 GAD items. The relative discreteness of the two sets of associations (i.e., SRS‐2, CASI‐4 GAD) is emphasized in Figure 2A.

There were negative associations between the SRS‐2 subscale for Social Awareness and CASI‐4 GAD items for *Difficulty controlling worry* (Control) and *Feeling worried* (Worry). Conversely, SRS‐2 Social Communication was positively associated with CASI‐GAD *Difficulty controlling worry*. Other nonsignificant associations were evident between SRS‐2 Social Motivation and the CASI‐4 GAD item about *Fatigue*; Social Cognition and *Feeling tense*; and RRB and *Feeling Restless* and having *Difficulties concentrating*.

## 4 Discussion

The first major finding from this research is the general confirmation of the discreteness of GAD and ASD symptomatology at a detailed level. Figures 1 and 2 show the separation of the major diagnostic criteria for these two disorders in an overall sense via the presence of two fairly discrete sets of connections. This confirmation of ASD and GAD as fairly discrete when using an alternative measure of ASD symptoms collected from parents rather than clinicians extends the previous findings reported by Montazeri et al. (2019). However, the detailed examination of the network between self‐reported GAD symptoms and five major aspects of ASD reported by parents extends the earlier work by Montazeri et al. (2019) in several key ways.

First, the use of a solely male sample allows for greater generalizability to autistic males than the previously used mixed‐sex sample reported by Montazeri et al. (2019). The age range in the present study is also wider than in Montazeri et al.'s (2019) sample. Second, the internal networks for SRS‐2 subscale scores and CASI‐4 GAD items represent relatively homogeneous constructs, each with their own ‘pathways' between symptoms. For example, the connections between SRS‐2 Social Communication and Social Cognition, Social Motivation, Social Awareness, and Restricted Interests and Repetitive Behaviours imply a possible pathway running from having an awareness of social situations, plus a motivation to engage socially, successfully interpreting social cues and then interacting socially with others. Because these SRS‐2 scores all imply deficits in these characteristics, this pathway reflects difficulties in each one, and perhaps an outcome that is in the form of behaviours that are likely to be described as involving repetitive behaviours (to reduce unwanted stress arousal). Similarly, there were significant positive networks between the CASI‐4 GAD scale items, most robustly between *Feeling Tense*, *Feeling Irritable* and *Being Unable to Control Worries*. A possible causal pathway is also evident here, most likely beginning with the major GAD symptom of being unable to control worries, leading to physiological tension that manifested itself in irritability towards others who may be perceived as representing a source of worry because of the inherent difficulty in managing social interactions that is part of the ASD symptomatology. It is also quite feasible that these feelings of physiological tension instigated the lack of sleep experienced by these boys and the resultant fatigue.

Third, there were three significant correlations between some of the two sets of items. Two of these were negative (Social Awareness: *Feeling worried*; *Having difficulty controlling my worries*), and one was positive (Social Communication: *Having difficulty controlling my worries*). The first two of these significant associations represented high SRS‐2 scores and low CASI‐4 GAD item scores, whereas the third significant association represented high SRS‐2 scores and high CASI‐4 GAD item scores. Because the high SRS‐2 score for Social Awareness equates to greater difficulty in being able to pick up on social cues, a tentative explanation for the first two significant associations may be that those autistic boys who had low levels of social awareness were not as worried (and therefore did not have difficulty controlling their worries) because they were not sufficiently socially able to pick up on social cues to be anxious about their difficulties in responding to those cues. Because none of the SRS‐2 subscale scores were significantly correlated with WASI‐II Full Scale IQ, that degree of (lack of) social awareness was not purely an effect due to inability to understand, but more likely a result of the neurological difficulties associated with autism per se. The third significant association (i.e., between Social Communication *and Having difficulty controlling my worries*) was positive, meaning that, as participants' difficulty in expressing social communication increased, so did their inability to control their worries. This association implies a desire to be able to communicate socially, an awareness of difficulty communicating and anxiety when that communication is not achieved.

This research holds important implications for clinical practice, particularly in the delivery of personalized medicine approaches to helping male autistic youth manage their social anxiety because these results emphasize the interaction between some aspects of ASD symptomatology and some symptoms of GAD, which may influence the delivery and effectiveness of standardized treatments. For example, the effectiveness of psychotherapy‐based treatments depends on the establishment of rapport between therapist and client (Constantino et al. 2020; Flückiger et al. 2018; Horvath and Luborsky 1993), but that process may be hindered by the interaction of GAD symptomatology regarding being unable to control worries and the presence of Social Communication difficulties in some autistic clients. Although cognitive behavioural therapy models have often been applied to treating anxiety in autistic youth (e.g., Wood et al. 2020), a meta‐analysis of 19 randomized control trials found that treatment effects were much reduced when followed up several weeks after treatment was completed (Sharma et al. 2021), suggestive of an immediate halo effect due to the attention and focus of the therapy setting rather than the treatment per se.

From those findings, plus the current results, it is apparent that applying a ‘one‐size‐fits‐all’ model of CBT or other standardized therapies could overlook the presence of associations between aspects of social communication and interaction difficulties and anxiety in some anxious youth. As such, there is a need to consider the relative strength of a client's SRS‐2 Social Communication and Social Awareness scores. If the former is a higher score than the latter, then effective therapy choices might focus upon how the high level of Social Communication difficulties (positively) impacts the client's difficulty in controlling their worries. If the client's scores on SRS‐2 Social Awareness are elevated, then these may be an indicator that therapy that is focused upon simply learning how to communicate with others might be less effective than working with the autistic client to emphasize the benefits of being able to respond to others' social cues.

### 4.1 Limitations and Strengths

Limitations include the cross‐sectional design of the study and the (planned) restriction of sampling to autistic males. The extension of these procedures to autistic females, plus the collection of data over time and from different ages of participants, will extend the generalizability of these results. The SRS‐2 and the CASI‐4 are well‐validated and reliable instruments, and the collection of GAD data from the autistic boys is a strength supported by previous studies that demonstrated parental bias when evaluating their autistic sons' anxiety. Network analysis provides a more reliable model of the intercorrelations between variables than is obtained from simple correlation matrices because it considers how a particular association fits within the entire range of associations being examined.

### 4.2 Conclusion

These results confirm that ASD and GAD are discrete at the symptom level, but also that they are connected in some specific ways that have not previously been reported via network analysis. Those connections are important considerations when planning individualized treatment for anxiety in young autistic males and may lead to more effective outcomes from the various therapies applied to this comorbidity.

## Author Contributions

All authors have made substantial contributions to the development of the research, data collection and analysis, and writing and have approved the final manuscript.

## Ethics Statement

This research was approved by the Bond University Human Research Ethics Committee in accordance with the Helsinki Declaration of 1964 (Approval No. RO1516, 15 September 2013).

## Consent

Written informed consent to participate was provided by the autistic boys' mothers, and the boys also gave written or verbal assent to participate, depending on their age.

## Conflicts of Interest

The authors declare no conflicts of interest.

## Data Availability

Data are available from the first author on request.
